# A cautionary tale of false-positive histoplasma urine antigen in an HIV patient: a case report

**DOI:** 10.1099/acmi.0.000929.v3

**Published:** 2025-05-28

**Authors:** Mohammad Z. Khrais, Jake Smith, Tanmay Gandhi, Shahrukh Arif, Juan Carlos Rico

**Affiliations:** 1 Department of Internal Medicine, University of Arkansas for Medical Sciences, Little Rock, AR, USA; 2 Division of Pulmonary Medicine, University of Arkansas for Medical Sciences, Little Rock, AR, USA; 3 Division of Infectious Diseases, University of Arkansas for Medical Sciences, Little Rock, AR, USA

**Keywords:** broncho-alveolar lavage, coccidioidomycosis, endobronchial ultrasound (EBUS), false positive, histoplasma antigen, human immunodeficiency virus

## Abstract

**Introduction.** Coccidioidomycosis, or Valley fever, is a fungal disease caused by *Coccidioides* species, prevalent in parts of the southwestern United States. It usually results from inhaling spores from soil and is a common cause of pneumonia in these regions.

**Case Presentation.** We present a unique case of coccidioidomycosis in an immunodeficient male patient secondary to human immunodeficiency virus infection with poor adherence to anti-retroviral treatment. After presenting with non-specific symptoms and pre-syncope, he was initially diagnosed with pneumonia based on chest X-ray findings, but his symptoms failed to improve with antibiotics. He was treated for presumed pulmonary histoplasmosis following a positive histoplasma urine antigen test. However, the patient worsened clinically. Following a computed tomography scan demonstrating a large necrotic lung consolidation, fungal stain and culture of tissue biopsied through endobronchial ultrasound confirmed coccidioidomycosis. The patient received 2 weeks of liposomal amphotericin with clinical improvement before discharge with itraconazole.

**Conclusion.** The histoplasma antigen test can be falsely positive due to cross-reaction with other fungal infections like blastomycosis, paracoccidioidomycosis or talaromycosis, and less frequently, coccidioidomycosis or aspergillosis. Diagnosis of coccidioidomycosis requires a high index of suspicion outside the expected geographic distribution in the appropriate clinical setting. Our case highlights the risk of false-positive antigen test results and the importance of invasive diagnostics, including bronchoscopy to obtain fungal cultures, if the diagnosis remains uncertain.

## Data Summary

This article was previously presented as an abstract at the 2023 CHEST Annual Scientific Meeting on 11 October 2023.

All data associated with this work is reported within the article.

## Introduction

Fungal infections in patients with human immunodeficiency virus (HIV) remain a significant cause of morbidity and mortality [1]. Histoplasmosis is a common infection among people with advanced HIV disease. It is caused by inhalation of the dimorphic fungus *Histoplasma capsulatum*, which is found in soil contaminated by bird or bat droppings. Histoplasmosis can manifest with non-specific symptoms like weight loss, fever and dyspnoea or can involve multiple organs in disseminated cases. Histoplasmosis is endemic in the United States, particularly in Ohio and Mississippi River Valleys [2].

Coccidioidomycosis is a less common endemic fungal infection caused by *Coccidioides* species and is transmitted via inhalation of fungal spores from soil. It can infect both immunocompetent and immunocompromised patients, presenting as a spectrum ranging from asymptomatic or mild, self-limiting respiratory illness to life-threatening pneumonia. Extra-pulmonary manifestations involving the skin, bones and central nervous system have also been reported [3]. Notably, most cases of coccidioidomycosis occur in the southwestern region of the United States (California, Arizona, Texas and Utah), with over 95% of cases diagnosed in Arizona and California [4,5].

## Case presentation

**Fig. 1.**
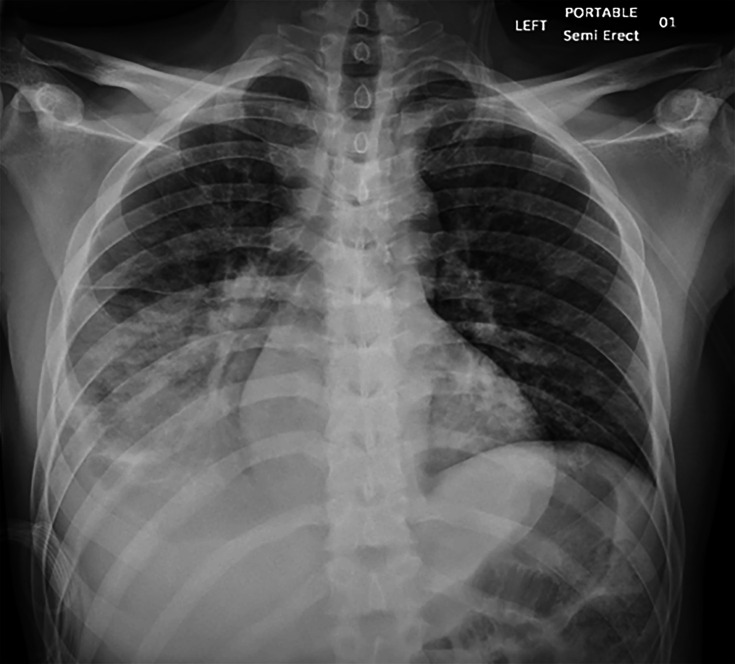
Chest X-ray from admission demonstrating right lower zone radiopacity with air bronchograms.

A male patient in his twenties with a medical history significant for congenital HIV, non-adherent to anti-retroviral treatment (ART) and cryptococcal meningitis presented to a rural hospital in Arkansas with fatigue and light-headedness. He was found to have a right lung opacity on chest X-ray (Fig. 1) and a positive urine histoplasma antigen test. However, the HIV workup indicated severe immunosuppression with a CD4 count of 4 cells µl^−1^ (Reference range: 430–1800 cells ul^−1^), and an HIV viral load of 75,432 copies ml^−1^ (Reference range: not detected). The patient was initially treated with amoxicillin–clavulanic acid for presumed bacterial pneumonia and fluconazole for presumed histoplasmosis.

His fever persisted, and his cough worsened. Three weeks after the initial presentation, he was transferred to our tertiary care centre. On presentation, he had a low-grade fever (maximum temperature of 100.5 °F), mild tachycardia (heart rate in the 100s-110s) and normal blood pressure and oxygen saturation on room air. Initial laboratory workup was significant for hypochromic anaemia with haemoglobin of 8 g dL^−1^, positive histoplasma and blastomyces antigen enzyme immunoassay (EIA) by MiraVista Diagnostics and a sputum fungal culture positive for one colony of *Blastomyces dermatitidis* (Table 1).

**Table 1.** Laboratory investigations

Test	Observed value	Reference range
CBC		
Haemoglobin	8.0 g dl^−1^	13.0–17.0 g dl^−1^
WBC	7.50 K µl^−1^	3.60–9.50 K µl^−1^
Neutrophils	69.9% – Absolute 5175 K µl^−1^	
Lymphocytes	5.3% – Absolute 397 K µl^−1^	
Eosinophils	0.0% – Absolute 0 K µl^−1^	
Platelets	532 K µl^−1^	150–450 K µl^−1^
LDH	262 IU l^−1^	100–248 IU l^−1^
Procalcitonin	0.05 ng ml^−1^	0.00–0.10 ng ml^−1^
Peripheral smear	Mild hypochromic anaemia	
Serum β-glucan	195 pg ml^−1^	<80 pg ml^−1^
Histoplasma antigens EIA	1.56 ng ml^−1^	None detected
Blastomyces antigens EIA	1.11 ng ml^−1^	None detected
COVID PCR	Negative	Negative
Screening MRSA PCR	Negative	Negative
Cryptococcal serum antigen	Negative	Negative
Aspergillus serum galactomannan	0.11	0.00–0.49
Histoplasma serum antibodies	Not detected	Not detected

LDH, lactate dehydrogenase; MRSA, methicillin-resistant *Staphylococcus aureus*; WBC, white blood cells.

**Fig. 2.**
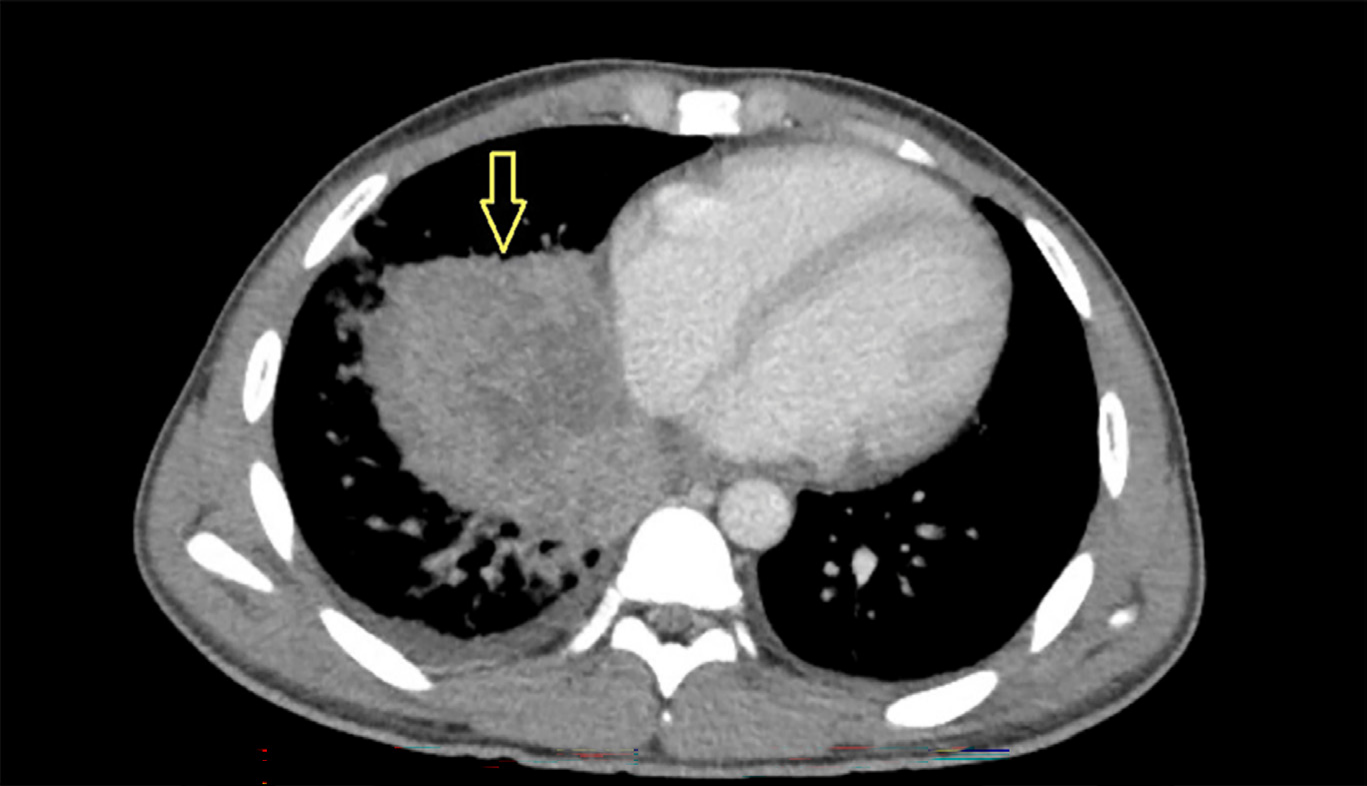
A CT scan with intravenous contrast demonstrating a necrotic lung lesion with small layering right-side pleural effusion.

Computed tomography (CT) of chest with contrast revealed a necrotic lung mass in the right lower lobe measuring 9×6.5×8.5 cm, with internal septation and cystic changes (Fig. 2). The patient underwent fine-needle aspiration guided by endobronchial ultrasound (EBUS) of the right lower lobe mass and bronchoalveolar lavage (BAL) of the right lower lobe. BAL showed 39% neutrophils and 60% macrophages. The β-glucan level was elevated in BAL fluid analysis. Definitive diagnosis with BAL and tissue cultures grew *Coccidioides immitis* and *Coccidioides posadasii*, establishing a geographically rare and unexpected diagnosis. The potato dextrose agar and the brain heart infusion cultures were used, and both were positive. After seeing the organism on calcofluor white stain, serology for coccidioidomycosis was obtained and further confirmed the diagnosis (Table 2).

**Table 2.** Coccidioidomycosis serology at the time of diagnosis and 1 year after diagnosis

Test	Observed value		Reference range
	At the time of diagnosis	One year after diagnosis	
Coccidioides IgM	3.7	0.7	<=0.9
Coccidioides IgG	1.2	11.1	<=0.9
ID band	Positive	Positive	Not detected
CF antibody	1 : 4	1 : 65536	<1 : 2

CF antibody, complement fixation antibody; ID band, immunodiffusion band.

**Fig. 3.**
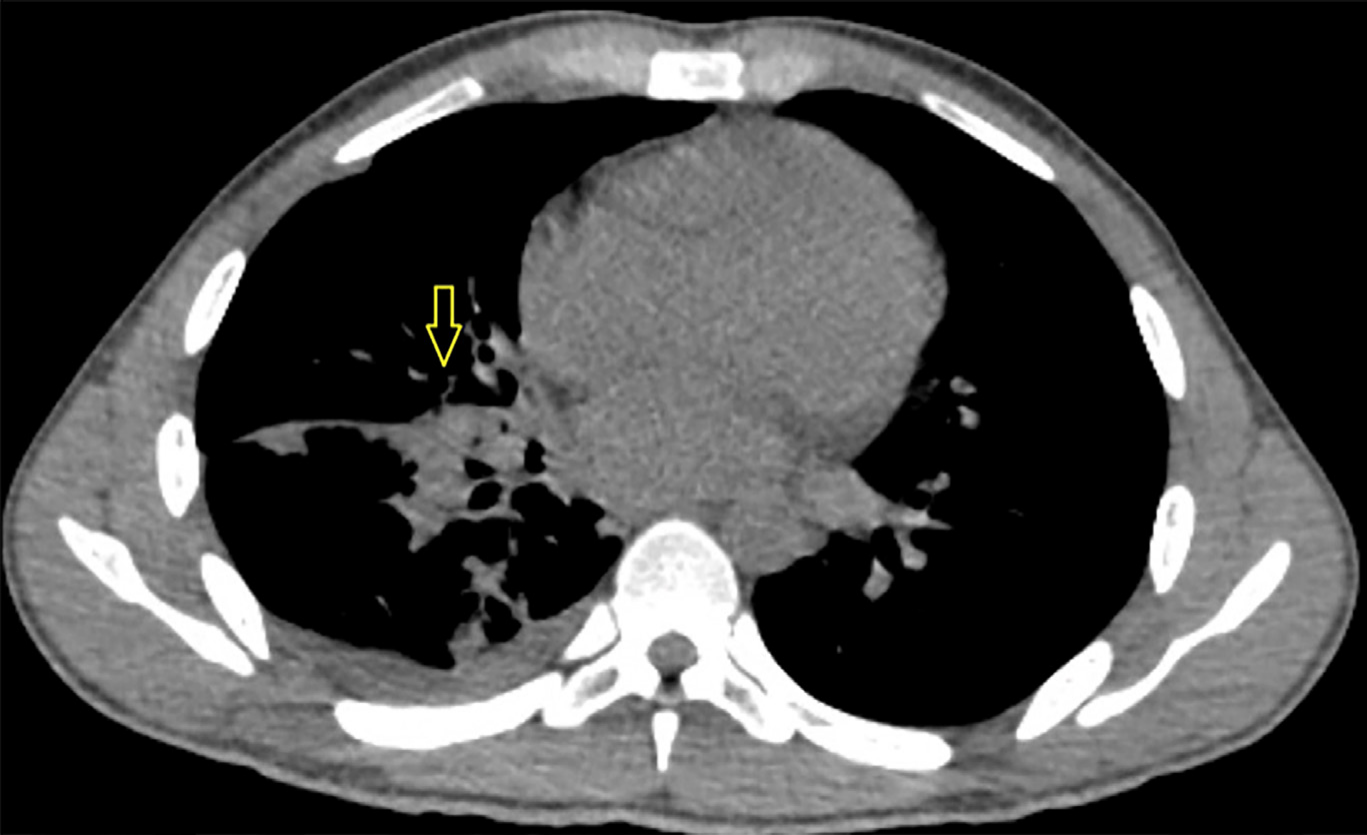
Repeat CT chest without contrast demonstrating decrease in size of right perihilar mass.

The patient was initially started on itraconazole but continued to have fevers with a maximum temperature of 105 °F and decreased oxygen saturation requiring supplemental oxygen via nasal cannula. Itraconazole was switched to intravenous (IV) liposomal amphotericin with a resolution of fever. The patient continued to improve and was discharged home on itraconazole after completing 14 days of IV liposomal amphotericin. The patient missed follow-up appointments after discharge and was readmitted a year later with cough and fever. He received itraconazole for presumed relapsed coccidioidomycosis, likely due to poor adherence to ART and itraconazole. A follow-up chest CT showed that the lung mass had decreased in size to 4.4×3.1 cm (Fig. 3).

## Discussion

Differentiating between histoplasmosis, blastomycosis or coccidioidomycosis can be challenging because these endemic mycoses often present with non-specific signs and symptoms. Additionally, high sensitivities and low specificities of their diagnostic tests can confuse diagnosis. This was particularly evident in our case, where the patient presented with non-specific symptoms, and both histoplasma and blastomyces antigen EIAs were positive on EIA. Furthermore, the patient’s non-invasive sputum culture grew a colony of *B. dermatitidis*. The MVista^®^ Quantitative Histoplasma Antigen EIA is a commercially available antigen test, which has 95–100 % sensitivity for histoplasma antigen in the urine [6]. Although antigen detection tests are most sensitive to diagnose histoplasmosis, they are prone to false-positive results due to cross-reactivity with other fungal infections such as blastomycosis, paracoccidioidomycosis, talaromycosis and aspergillosis, and even medications such as rabbit anti-thymocyte globulin infusions [7,10]. This cross-reactivity is due to galactomannan and other polysaccharides in the cell walls of different mycotic species, and those epitopes can be recognized by the same antibodies used in the antigen tests. In one study, histoplasma antigen tests were suggested as a potential diagnostic tool for coccidioidomycosis, as 11 of 19 (58%) patients with coccidioidomycosis had a positive histoplasma antigen test [11].

Traditional methods for detecting coccidioidomycosis, such as culture and direct visualization, have limitations and pose risks. KOH (potassium hydroxide) and Silver Stain are preferred for their simplicity and affordability in direct observation. Coccidioides antigen detection tests exist, but their sensitivity is lower than histoplasma antigen tests to detect Coccidioides. However, antigen detection using urine samples is non-invasive and appears more sensitive than serum or BAL. While antigen and antibody tests play important roles in clinical settings, they may be insufficient on their own [12]. Serology is a more reliable and commonly used test to diagnose coccidioidomycosis. Combining antigen and antibody EIA tests improves detection accuracy, and the measurement of combined IgM and IgG by EIA can increase sensitivity to 95% or higher [10]. Although culture or direct visualization is the gold standard for the diagnosis of coccidioidomycosis, its utility is limited by lower sensitivity and extended incubation times [13]. The GeneSTAT Coccidioides Assay is the first FDA-authorized real-time PCR-based test for the rapid detection of coccidioidomycosis, complementing serology and antigen detection to enhance diagnostic accuracy [14].

This case report highlights the importance of considering diagnoses even beyond commonly accepted geographic boundaries. While endemic mycoses have been historically named after their approximate geographic distribution, their ranges can be broader than their names suggest, leading to incorrect or missed diagnoses [15]. Our patient was diagnosed in an area with a high incidence of histoplasmosis and outside of the endemic geographic range of coccidioidomycosis, with minimal incidence of coccidioidomycosis (less than 5 cases per 100,000 people) [16,17]. Furthermore, patients travel. Our patient made frequent trips to Texas for work, which actually may have been the location of his exposure.

Coinfection with different endemic mycoses should be considered, especially in immunocompromised patients. In the case of our patient, it remains unclear whether the single *B. dermatitidis* colony that grew from the sputum culture was misidentified, was correctly identified but did not contribute to the patient’s pathology or contributed to his pathology but was treated effectively by the itraconazole prescribed for Coccidioides. This case highlights the potential consequences of relying solely on antigen tests, as exemplified by documented cases of delayed diagnosis, prolonged symptoms and possible unnecessary antibacterial treatment in patients with coccidioidomycosis diagnosed outside endemic areas [17,18]. Finally, BAL and EBUS should be considered when diagnosis is uncertain.

## Abbreviations

ART: anti-retroviral treatment
CT: computed tomography
EBUS: endobronchial ultrasound
HIV: human immunodeficiency virus
IV: intravenous
